# The Influence of Herd Mentality on Rating Bias and Popularity Bias: A Bi-Process Debiasing Recommendation Model Based on Matrix Factorization

**DOI:** 10.3390/bs13010063

**Published:** 2023-01-10

**Authors:** Xinjie Su, Peng Li, Xinru Zhu

**Affiliations:** School of Management, Harbin University of Commerce, Harbin 150028, China

**Keywords:** power-law distribution, herd mentality, rating bias, popularity bias, recommender system

## Abstract

To reduce the impact of rating bias and popularity bias in recommender system, and make the recommender system reach a balance between recommendation utility and debias effect at the same time, we propose a bi-process debiasing recommendation model based on matrix factorization. Firstly, considering the problem that the user’s ratings are affected by the herd mentality, which leads to a consistency between the rating and the selection of rating items, resulting in the power-law distribution, the k-times parabolic fuzzy distribution was used to fuse the user’s age to redistribute the ratings. Secondly, the loss function is optimized by the continuously increasing flow and popularity of items. Finally, user emotion and item popularity are combined to construct user psychological tendency, which is divided into three levels: strong, medium and weak, and different levels are given different weights. To verify the performance of the model, the experimental results on real datasets show that the model proposed in this paper not only effectively reduces the recommendation bias but also ensures the recommendation utility.

## 1. Introduction

As an important tool to alleviate information overload, the recommender system makes a significant contribution to improving personalized experiences such as e-commerce shopping, movie recommendations, and travel recommendations by using historical interaction data between users and items to generate recommendation predictions [1,2]. However, the recommender systems that only aims to improve the recommendation utility can easily lead to the Matthew effect [3], Filter bubble [4], Process Fairness [5], Outcome Fairness [5], and other problems, and the bias of the recommender systems is one of the reasons for increasing the unfairness of process and unfairness of outcome [6]. Recommender bias includes popularity bias, exposure bias, position bias, rating bias, etc., which are commonly found in data, models, and results [7]. Influenced by the herd mentality, the rating bias is manifested in that users tend to make similar ratings with others or choose similar rating items, even if the rating or selection is contrary to the original intention of users, so the rating bias fails to reflect the real preferences of users [8]. When the ratings of the user group all focus on the same item, the item becomes a high-popularity item. The recommendation model was trained with this kind of user-item historical interaction information, which makes the prediction results contain popularity bias. Popularity bias refers to the fact that items with high popularity are recommended more often than their popularity [9]. Popularity bias has an important impact on the data, model, and results of the recommender systems, and is one of the reasons why most items are not fairly recommended [2].

From the perspective of data, the data presents a power-law distribution [10], that is, in most real-world datasets, the number of high-popularity items is much less than that of low-popularity ones. However, the corresponding attention and flow are very different from the amount, that is, a small number of high-popularity items get a large number of user visits and flow, while a large number of low-popularity items share a small amount of flow from users. Therefore, the distribution of data shows a power-law distribution with a high degree of imbalance, which affects the recommendation model and results.

For models, the collaborative filtering recommendation models based on matrix factorization tend to expand the bias by over-recommending items with high popularity [10]. Since the purpose of the recommendation model is to predict the rating value of unrated items by users using the historical interaction data between users and items, its goal is to continuously fit the data to achieve the minimum loss, so as to achieve accurate prediction. It is precisely because of the power-law distribution of data and the blind fitting of the model that the existing popularity bias in the data source is further amplified with the training of the model, and then in the subsequent recommendation, the system still tends to recommend the items with high popularity with high frequency.

From the perspective of results, popularity bias damages user satisfaction and trust in recommendation services [11]. If the results with popularity bias are recommended to users, the information received by users will be homogenized. In the long run, users are prone to burnout and have aesthetic fatigue, the system will lose users, and users will also reduce their trust in the recommendation service. Therefore, it is undoubtedly critical to mitigating popularity bias from the perspective of data, models, or results.

In view of this, this paper combines the data and model perspectives, respectively, considering the power-law distribution phenomenon of user ratings affected by herd mentality and the problem that the recommendation model based on matrix factorization will amplify the popularity bias. Starting from the data and model, in-processing and post-processing are optimized in the recommendation cycle to reduce the impact of bias on the results, in order to achieve a balance between unbiased recommendation and improved accuracy.

At the same time, this paper takes herd mentality as the entry point to explore the impact of users’ rating choices on the rating bias and popularity bias, and proposes the corresponding debiasing model to effectively mitigate the bias. In addition, according to the research results, this paper extracts the important factors affecting commodity sales and user satisfaction, and puts forward corresponding suggestions to the platform and merchants.

### 1.1. Herd Mentality

Psychological research shows that the herd mentality of users is directly related to its decision-making behavior [12]. In the context of group behavior, people underestimate their judgments and individuals imitate group choices [13]. Especially when users are in an uncertain environment, imitation is a “safe” choice that users can make. However, this choice is not necessarily subjective, which is different from conformity [14]. Conformity behavior depends on the “observation” of others’ behavior, and is more a kind of “following”. Subjectivity, on the other hand, often relies on information received from important people [15,16].

### 1.2. Rating Bias

Liu et al. [8] believed that influenced by the high rating of a project’s public comments, users are likely to change their original low rating in order to avoid a harsh rating. This kind of conformity phenomenon is common, which will lead to the bias of user ratings. Krishnan et al. [17] believed that when users rated items before or after being exposed to public opinion, user evaluations followed different distributions. In addition, Chaney et al. [18] and Wang et al. [19] showed that conformity bias may be caused by social influence, in which users tend to behave similarly to their friends. Thus, the observed ratings are biased and may not reflect a user’s true preference for the item. Adomavicius et al. [20] showed that if the user preference rating is distorted, it will pollute the user’s subsequent input rating on the recommender system, and further cause the uncertainty of the recommender system, so as to provide users with fuzzy views of non-real preferences. Xu et al. [1] believed that the observed ratings would lead to redundant or inaccurate recommendation results for all users. Therefore, Xu et al. [1] aimed to explore the hidden information of observation ratings to alleviate this recommendation dilemma.

### 1.3. Popularity Bias

Popularity bias results in users who tend to evaluate popular items, resulting in the majority of user evaluations clustered in popular items, while the evaluation of long-tail items are ignored. In addition, the system will recommend similar items to users according to their frequent clicking behavior, and the Matthew effect will appear in such a cycle, thus affecting the real preferences of users, resulting in a decline in user satisfaction and content richness. Liu et al. [21] argued that the feedback loop ecology of recommender systems further exacerbated this Matthew effect. Jadidinejad et al. [22] pointed out that recommender systems are usually evaluated based on user interactions collected from existing and deployed recommender systems. As a result, users only provide feedback on the published project, creating a closed-loop feedback. The feedback loop ecology of the recommender system further intensifies this Matthew effect. Mansoury et al. [2] pointed out that one of the main reasons why different items do not receive fair exposure in recommendations is the influence of popularity bias, that is, a few popular items are over-recommended, while most other items do not receive due attention. Abdollahpouri et al. [23] also showed that this bias towards popular items will have a negative impact on less popular items and new items in the system. Jannach et al. [24] believed that the most advanced recommendation models also show obvious bias from the recommended items favored by most people. Saito et al. [25] believed that popular items attract more attention than other items, so popular items can receive more user behaviors. These popular items will have a greater impact on model training, making the model recommendation results more favorable to these items.

### 1.4. Related Research from the Perspective of Data

From the perspective of data, the historical interaction data between users and items are mainly composed of rating information by users. Sreepada and Patra [26] verified that rating datasets commonly used in recommender systems follow power-law distribution. One of the reasons for the power-law distribution is that user ratings are easily affected by external factors, including but not limited to herd mentality, social influence bias, and persuasion bias, which tends to make the ratings consistent and centralized. Moreover, it leads to the polarization of the scoring situation of high-popularity items and low-popularity items, and the low-popularity items are increasingly marginalized. Liu et al. [8] believed that users will be influenced by others’ opinions when making choices online. Sipos et al. [27] concluded from an experiment on voting that users’ behaviors are not always honest, and their decisions are largely derived from the surrounding environment. This phenomenon of user ratings being changed by herd mentality exists in most scenarios, including programs and digital products [28,29,30].

Some related studies use matrix factorization to indirectly improve the bias of users influenced by others. Chaney et al. [18] developed the social Poisson decomposition based on the Bayesian model, which uses the user’s potential preferences and the potential influence of social relations to explain the user’s consumption behavior on the item at the social level. Wang et al. [19] proposed a personalized social association preference matrix factorization model based on probability matrix factorization considering the influence of strong social ties and weak social ties on users. There are also related studies that directly use existing resources to improve. Sreepada and Patra [31] proposed a hybrid framework to mitigate the long-tail effect by using the Siamese network and reformulating the input of the network. Steck [32] adopted the method of data rescoring to increase the rating of long-tail items. Meanwhile, Sreepada and Patra [26] injected ratings into long-tail items in a systematic way to provide a new perspective for solving long-tail problems.

### 1.5. Related Research from the Perspective of the Model

From the perspective of the model, as a common explicit factor in recommender systems, rating data are often the preferred input of the model because it is easy to obtain and contains obvious user preferences to a certain extent. However, the model is easy to amplify the inherent bias in the data and even brings other recommendation biases. For example, the collaborative filtering recommendation model based on matrix factorization tends to amplify the popularity bias. Liu et al. [33] showed that ignoring the bias would lead to the recommendation model converging into a biased suboptimal solution. Mena-Maldonado et al. [34] pointed out that the main goal of the recommender system is to recommend users’ favorite items rather than popular items. However, recommender systems themselves set up a feedback loop, and Carraro and Bridge [35] pointed out that users are generally more likely to interact with the suggestions provided by the system than with other items.

Some related studies tend to quantify the popularity bias in the in-processing stage of the recommendation life cycle and make corresponding optimization strategies. Bhadani [36] quantified the popularity bias by using the existing market data, deepened the understanding of the popularity bias and promoted the stable development of the recommender system. Steck [32] adopted the method of weight allocation, aiming to increase the weight of long-tail items. Some studies also adopted a new scoring strategy in the post-processing stage of the recommendation life cycle, aiming to improve the recommendation of low-ranked items in line with user needs. Zhu et al. [37] combined user value scale and preference degree to compensate low-popularity items to improve their probability of being recommended. Abdollahpouri et al. [38] designed a post-processing framework based on diversified re-ranking, which is flexibly applicable to the output of the recommender system and increases the proportion of low-popularity items in the recommendation.

In summary, most scholars focus on one perspective of the recommender system or are committed to solving a type of bias in the recommender system, lacking the universal ability to consider mixed data and model bias. From the perspective of the whole life cycle of the recommender system, both data and model play a decisive role in the results.

### 1.6. Contribution of This Paper

Therefore, this paper proposes a bi-process debiasing model that mixes rating bias and popularity bias from the perspective of data and model. The main contributions of this paper are as follows:

1. By taking the rating bias in the data as an entry point, considering that the user’s rating behavior can easily be affected by herd mentality, and integrating the characteristics of different user age groups, the K-times parabolic fuzzy distribution is used to adjust the user’s historical rating information to reduce the rating bias.
2. With the popularity bias in the model as the starting point, the continuously increasing flow and popularity of the item are considered, and by incorporating the debiased-rating as the weight to optimize the model, the scoring bias and popularity bias are reduced.
3. The psychological line is introduced as a proxy tool for studying user emotions, and the popularity index is introduced as a proxy tool for item popularity. The psychological tendencies of users are divided into three levels: strong, medium, and weak, and different weighting strategies are adopted for different levels to ensure the balance between recommendation utility and debias effect.

## 2. Materials and Methods

### 2.1. Preliminaries

#### 2.1.1. Psychological Line

The purpose of psychological line (PSY) in the stock market is to explore the psychological fluctuations of investors on the rise and fall of the stock market, which can reflect the strength of investors’ willingness to buy and sell, and is one of the emotional indicators for the study of investor psychology. The calculation formula of the psychological line is as follows:

(1) $$PSY=\frac{N\_rise}{N}*100\%$$

where, N represents the number of days, which is permanently set at 12 in the stock market application; N_rise indicates the number of days in N in which the stock market rises.

The stock rises continuously, and the investor strongly invests in the willingness to buy this stock. In recommender systems, the continuous increase of browsing flow leads to an item’s high popularity, which will affect the user in selecting the item. At the same time, system suggestions are more inclined to recommend items with high popularity, which forms a bad closed-loop feedback. However, this herd mentality and frequent browsing of similar types of highly popular items are not permanent. With the passage of time or repeated push, users’ emotions will change significantly, leading to the birth of reverse psychology, further affecting the benefits of the item and the platform, and more seriously, leading to the loss of a large number of users in the platform. Therefore, this paper creatively applies psychological lines to the recommender system as one of the tools for studying user emotion agents.

#### 2.1.2. Sentiment Indicators

Sentiment indicators (AR) reflect the sentiment of market trading in the stock market, attach importance to the opening price of the stock market, and reflect the market situation and stock price trend through the opening price of a certain period. The sentiment indicator is calculated using the following formula:

(2) $$AR=\frac{\sum high-open}{\sum open-low}*100\%$$

where high represents the highest price of the stock in a fixed period; low indicates the lowest price of a stock in a fixed period; open indicates the opening price of a stock in a fixed period. In stock market applications, the fixed period is usually set to 26 days.

When the market sentiment is high, the stock price will do better, but too high means that the price may fall at any time. In the recommender system, the higher the popularity of the item, the easier it is to attract the attention of users. Although high-popularity items are helpful to increase system flow and guide user behavior, popularity bias occurs when high-popularity items are recommended more frequently than their popularity, which makes long-tail items that are low-popularity items difficult to recommend. This will have adverse effects on recommendation platforms, suppliers, and users in the long run. Therefore, this paper attaches importance to the average popularity of all items in the system and creatively applies the sentiment indicators to the recommender system as one of the proxy tools to study the popularity of items.

#### 2.1.3. K-Order Parabolic Fuzzy Distribution

The fuzzy distribution [39] has certain advantages in dealing with uncertain information, especially for multi-attribute decision-making problems. In most cases, the result of the decision is not only black and white, as sometimes the result will appear to be close to one side or ambiguous. However, fuzzy does not mean that it is an incorrect state; fuzzy distribution is the condensation of fuzzy state, so that it forms a tangible concept. The calculation formula of k-order parabolic fuzzy distribution is as follows:

(3) $$\mu_{A}=\left\{ \begin{array}{l} {\left(\frac{x-a}{b-a}\right)}^{k},a\leq x\leq b\\ 1,b\leq x\leq c\\ {\left(\frac{d-x}{d-c}\right)}^{k},c\leq x\leq d\\ 0,x<a,x\geq d \end{array}\right.$$

where, the fuzzy set A is determined by any mapping from the domain X to the closed interval [0, 1] and A=(a,b,c,d) is the fuzzy number on the real number R; $\mu_{A}$ is the membership function of the fuzzy set A. k denotes the degree of parabolic fuzzy distribution.

### 2.2. Model Building

#### 2.2.1. Similarity Measurement Model Based on K-Order Parabolic Fuzzy Distribution

First, consider that users of the same age group are more likely to have the same preferences and rating habits. The purpose of age grouping is to bring active users as close as possible to a group of neighbors [40], but the distribution of age has no natural boundary in classical set theory [41]. However, human interpretation allows a gradual transition between the categories of “old” and “too old” [42]. Therefore, combined with the age distribution of users in the real dataset, this paper divides the users into three age groups, which are group A: (1–30), group B: (15–60), and group C: (45–75). However, age is a user attribute with ambiguous nature, that is, an exact age value, such as 30 years old, can be classified as young users or middle-aged users. At the same time, the user’s age and the user’s behavior sometimes do not match, such as “an old head on young shoulders”.

Secondly, because the user’s rating behavior is easily affected by herd mentality, the rating information may not conform to the user’s real preference. According to the common scoring mechanism of 1–5 points, this paper divides the user’s rating of the item into three groups: group D: (0–2), group E: (1–4), and group F: (3–5). However, the evaluation of 1 to 5 points is a kind of rating with ambiguity, that is, when the rating tends to the middle rating, the system cannot well capture whether the user’s preference for the item tends to be good or bad. At the same time, users are influenced by the herd mentality, which makes their ratings consistent with the surrounding crowd, and also makes the ratings fuzzy.

Given this, group A has intersecting parts with group B, group B has intersecting parts with group C, group D has intersecting parts with group E, and group E has intersecting parts with group F, to reflect the real situation in line with the real problem.

For group A, a = 0, b = 0, c = 15, d = 30; for group B, a = 15, b = 30, c = 45, d = 60; for group C, a = 45, b = 60, c = 75, d = 75; for group D, a = 0, b = 0, c = 1, d = 2; for group E, a = 1, b = 2, c = 3, d = 4; for group F, a = 3, b = 4, c = 5, and d = 5. Let k = 1 and transform it into first-order parabolic fuzzy distribution.

The membership function of groups A, B, C are as follows:

(4) $$A_{\mu_{A}}(x)=\left\{ \begin{array}{l} 1,0\leq x\leq 15\\ (30-x)/15,15\leq x\leq 30\\ 0,x<0,x\geq 30 \end{array}\right.$$

(5) $$B_{\mu_{A}}(x)=\left\{ \begin{array}{l} (x-15)/15,15\leq x\leq 30\\ 1,30\leq x\leq 45\\ (60-x)/15,45\leq x\leq 60\\ 0,x<15,x\geq 60 \end{array}\right.$$

(6) $$C_{\mu_{A}}(x)=\left\{ \begin{array}{l} (x-45)/15,45\leq x\leq 60\\ 1,60\leq x\leq 75\\ 0,x<45,x\geq 75 \end{array}\right.$$

The membership function of groups D, E, F are as follows:

(7) $$D_{\mu_{A}}(x)=\left\{ \begin{array}{l} 1,0\leq x\leq 1\\ -x,1\leq x\leq 2\\ 0,x<0,x\geq 2 \end{array}\right.$$

(8) $$E_{\mu_{A}}(x)=\left\{ \begin{array}{l} x-1,1\leq x\leq 2\\ 1,2\leq x\leq 3\\ 4-x,3\leq x\leq 4\\ 0,x<1,x\geq 4 \end{array}\right.$$

(9) $$F_{\mu_{A}}(x)=\left\{ \begin{array}{l} x-3,3\leq x\leq 4\\ 1,3\leq x\leq 4\\ 0,x<3,x\geq 5 \end{array}\right.$$

Finally, because the improved Euclidean distance function has good performance in compatibility with multi-attribute similarity calculation, the fuzzy distance function proposed by Kant and Bharadwaj [43] is used to calculate the similar user preference rating of a parabolic fuzzy distribution integrating user age and rating information. The formula of fuzzy distance function is as follows:

(10) $$Fone(u,v)=\frac{1}{3}\sum_{j=1}^{3}\sqrt{{\left(u_{i,j}-v_{i,j}\right)}^{2}}$$

(11) $$Fsim(u,v)=1-\frac{1}{2}\sum_{i=1}^{2}Fone(u,v)$$

where, $u_{i,j}$ and $v_{i,j}$ respectively represent the corresponding membership degree values of the j-th group when user u and v take i as the scoring information or age information. Fone(u,v) represents the fuzzy distance function of single information.

By fusing the user’s age and rating information, it was converted into a k-order parabolic fuzzy distribution, and the fuzzy distance function between users based on this distribution was calculated to obtain the similar user set. Then, the prediction function was used to calculate the predicted rating of the user for the item, which was used as the weight of the debiased rating, and the weight matrix of the debiased rating was denoted as w. The prediction function is as follows:

(12) $$w_{u,t}=\bar{r_{u}}+\frac{\sum_{v\in V}Fsim(u,v)*(r_{v,t}-\bar{r_{v}})}{\sum_{v\in V}Fsim(u,v)}$$

where, V represents the set of users with similar feature preferences; $r_{v,t}$ represents the actual rating of the item t by user v; $\bar{r_{u}}$ and $\bar{r_{v}}$ represents the average ratings of user u and v, respectively.

#### 2.2.2. Loss Function Based on Continuously Increasing Flow and Popularity

The continuously increasing flow and popularity of items are the key points to explore the user sentiment and popularity of the item, which further affects the user’s rating decision. Firstly, the weight matrix of the debiased rating is normalized, and the processing formula is as follows:

(13) $$nw_{ut}=\frac{w_{ut}-min_{\begin{array}{l} 1\leq u\leq U\\ 1\leq t\leq T \end{array}}\{w_{ut}\}}{max_{\begin{array}{l} 1\leq u\leq U\\ 1\leq t\leq T \end{array}}\{w_{ut}\}-min_{\begin{array}{l} 1\leq u\leq U\\ 1\leq t\leq T \end{array}}\{w_{ut}\}}$$

where U represents the set of users; T represents the set of items.

Secondly, the debiased rating normalized by Equation (13) is used as the weight. Finally, the matrix factorization model obtained by integrating the continuously increasing flow and popularity of the item is as follows:

(14) $$\begin{array}{l} ℒ=nw_{ut}{\left(r_{ut}-\sum_{k=1}^{k}p_{uk}*q_{kt}\right)}^{2}+\frac{\lambda }{2}({\left\|p_{uk}+w_{uk}\right\|}^{2}+{\left\|q_{kt}+w_{kt}\right\|}^{2})+\frac{\lambda_{1}+\lambda_{2}}{2}{\left\|p_{uk}+\frac{1}{mcount_{k}}+mdate_{k}\right\|}^{2}\\ +\frac{\lambda_{1}+\lambda_{2}}{2}{\left\|q_{kt}+\frac{1}{mcount_{k}}+mdate_{k}\right\|}^{2}+\frac{\lambda_{3}}{2}{\left\|mcount\right\|}^{2}+\frac{\lambda_{4}}{2}{\left\|mdate\right\|}^{2} \end{array}$$

where, k represents the dimension of hidden factor space; $r_{ut}$ represents the rating of item t by user u; p and q represent k dimensional user latent factor matrix and dimensional item latent factor matrix, respectively; $mdate_{k}$ and $mcount_{k}$ represent the continuously increasing flow and popularity of the item in k dimension, respectively; mdate and mcount represent successively increasing flow value sequence and popularity value sequence of all items, respectively. $\lambda ,\lambda_{1},\lambda_{2},\lambda_{3},\lambda_{4}$ represent the regularization parameters.

#### 2.2.3. Recommendation Model Based on User Emotion and Item Popularity

User sentiment and item popularity are important factors affecting user rating decisions. In the initial case, users will be affected by herd mentality and persuasion bias, and follow the crowd to browse the highly popular items, resulting in the vast majority of flow converging on the highly popular items. However, with the increase of users’ historical behavior information, the recommendation model will predict users’ clicking behavior based on it. The model takes existing historical interaction data as input and a list of suggestions as output. Over time, the list of suggestions will be highly consistent with the user’s history, but this scenario is just a stereotypical information prediction and does not take into account the user’s emotional changes. The system recommends items with high consistency for a long time, which will cause users to become bored and even more frustrated with the platform. At the same time, the popularity of the item is the key to guiding the user’s behavior. In the initial state, users tend to browse the popular items in the vast number of items, and a series of interaction records are generated. However, with the formation of closed-loop feedback, high-popularity items are recommended more than their popularity, which brings adverse effects.

Given this, this paper constructs the user emotion evaluation function based on the psychological line. The larger the value, the more positive the user’s emotion is and the more inclined the user is to give a higher rating to the recommended item. The user emotion evaluation function is as follows:

(15) $$emotion(u)=\frac{mdate(t)}{N}$$

where, mdate(t) represents the maximum continuously increasing flow of item t in time period N.

At the same time, in the study of item popularity, we focus on the average popularity of all items in the system. Therefore, the item popularity evaluation function is constructed based on the sentiment indicators. The larger the value is, the higher the item popularity is, and the more users are inclined to interact with the recommended item. The item popularity evaluation function is as follows:

(16) $$popularity(t)=\frac{mcount(t)-\bar{g}}{\bar{g}-icount(t)}$$

where, mcount(t) and icount(t) represent the maximum and minimum popularity value of the item, respectively; $\bar{g}$ represents the average popularity of all items in the system.

According to the strong and weak tendency of user emotion and item popularity, the psychological tendency function is constructed. The psychological tendency corresponds to the degree to which users will interact with the item recommended by the system and give higher ratings. The psychological tendency function is as follows:

(17) $$\theta_{ut}=emotion_{ut}+popularity_{ut}$$

The psychological tendency values were divided into three levels: strong (6, 9], medium (3, 6], and weak [0, 3]. Different weight allocation strategies are adopted when the user psychology is in different level intervals, and the final model prediction rating is as follows:

(18) $${\hat{r}}_{ut}=\left\{ \begin{array}{l} e^{\alpha }*({\hat{r}}_{ut}+\frac{1}{mcount_{t}}),6<\theta \leq 9\\ e^{\beta }*({\hat{r}}_{ut}+\frac{1}{mcount_{t}}),3<\theta \leq 6\\ e^{\gamma }*({\hat{r}}_{ut}+\frac{1}{mcount_{t}}),0\leq \theta \leq 3 \end{array}\right.$$

where, α ,β ,γ is the weight parameter of strong, medium, weak, and psychological tendency, respectively, and α+β+γ=1.

## 3. Results

### 3.1. Experimental Preparation

#### 3.1.1. Experimental Dataset

Movielens dataset [44] is widely used in recommender systems. It contains user-item rating, user occupation, user gender, user age, and other information, and is one of the famous recommender datasets. Meanwhile, Sreepada and Patra [12] have verified that the rating data in Movielens follows the power-law distribution, which meets the experimental requirements of this paper. In this paper, the ratio of training set: validation set: test set is 7:2:1. The information about the dataset is shown in Table 1.

#### 3.1.2. Evaluation Metrics

To evaluate the performance of the model from two levels of recommendation utility and recommendation bias, this paper uses two types of metrics to evaluate the proposed model.

1. Recommendation utility

(19) $$NDCG^{@}s=\frac{DCG^{@}s}{IDCG^{@}s}$$

(20) $$DCG^{@}s=\sum_{i=1}^{s}\frac{2^{rel_{i}}-1}{\log_{2}(i+1)}$$

NDCG is one of the commonly used evaluation metrics of recommendation utility. The higher the value of NDCG, the better the recommendation utility of the model. Where, ${\mathrm{rel}}_{i}$ represents the true relevance of item i; s represents the recommended number. $IDCG^{@}s$ is $DCG^{@}s$ in the ideal state.

2. Recommendation bias

(21) $$\mathrm{PRU}=-\frac{1}{N}\sum_{u\in U}SCC(\mathrm{pop}(I_{s}),\mathrm{pre}({\hat{I}}_{s}))$$

PRU [37] measures the popularity bias from the perspective of users. The smaller the value of PRU is, the smaller the popularity bias of the model from the perspective of users is. Where, $I_{s}$ represents the collection of historical items; ${\hat{I}}_{s}$ represents the set of predicted items; N represents the total number of items; SCC(⋅,⋅) represents the calculated Spearman correlation coefficient of the two; $\mathrm{pop}(I_{s})$ represents the popularity list of historical items; $\mathrm{pre}({\hat{I}}_{s})$ represents the ranking list of recommended items predicted by the model.

(22) $$D\_M=\frac{1}{D(h{\left(I_{s}\right)}^{@}l)}*(D(p{\left({\hat{I}}_{s}\right)}^{@}l)-D(h{\left(I_{s}\right)}^{@}l))$$

D_M [45] measured the difference in popularity distribution between the historical item list and the item recommendation list predicted by the model from the five dimensions of mean, median, variance, skew, and kurtosis of the data, denoted as D_Mean, D_Median, D_Var, D_Skew, and D_Kurtosis, respectively. Where, $h{\left(I_{s}\right)}^{@}l$ represents the popularity list of historical items of length l; $p{\left(I_{s}\right)}^{@}l$ represents the list of recommended item popularity predicted by the model with length l; D(.) means mean, median, variance, skew, and kurtosis as measures.

When D(.) is chosen as the mean and median, if D_Mean or D_Median is positive, it means that the recommendation model recommends more popular items to users on the whole. When D(.) is the variance, if D_Var is positive, it means that the list of recommended items predicted by the model is more diverse than the user’s historical items. When D(.) is skew, if D_Skew is positive, it means that the right tail of the distribution of the recommendation list predicted by the model is heavier than the tail of the distribution of the user’s historical items relative to the left tail. When D(.) is kurtosis, if D_ Kurtosis is positive, it means that the recommendation distribution is close to the normal distribution to some extent. The tail of the popularity distribution of recommended items predicted by the model is heavier than its corresponding items.

In general, when D(.) is the mean, median, and variance, D_M tends to evaluate the recommendation of the model for items with high popularity. When D(.) is skew and kurtosis, D_M tends to evaluate the recommendation of the model for long-tail items, that is, low-prevalence items.

### 3.2. Psychological Tendency Parameter Settings

Since the frequency of all popular items being recommended does not necessarily exceed their popularity, the medium-popularity items may not cause popularity bias, and blindly reducing the proportion of high-popularity items will actually harm the recommendation accuracy. At the same time, in the composition of psychological tendency function, the calculation of user sentiment and item popularity is affected by the popularity of the item, and the strength of psychological tendency is positively correlated with the popularity. Therefore, this paper does not consider increasing the weight of the weak psychological tendency interval in the setting of psychological tendency parameters, and focuses on the recommendation utility and recommendation bias when users are in the strong psychological tendency interval. The step of parameter selection is set to 0.1, and the experimental results are shown in Table 2.

According to Table 2, when α is maximized, the recommendation utility reaches the optimum, but the recommendation bias also reaches the maximum. On the contrary, when γ takes 0.4, the maximum value of low weight, it means that compared with other low-weight values, the weight of low-popular items in the weak psychological tendency interval is increased, so the recommendation bias is the smallest and the debias effect is the best. However, if the weight is excessively increased, the recommendation utility will be lost. When β takes the maximum value of the low weight, the essence is that it increases the weight of medium-popular items, so its recommendation bias is between the bias when α takes the maximum value and the bias when γ takes the maximum value. At the same time, since the user-item interaction information in the medium psychological tendency interval has the largest amount among the strong, medium, and weak intervals, its recommendation utility is not as good as the recommendation utility when γ is the maximum.

Based on the above situation, in order to balance the weights of strong, medium, and weak intervals, and make the model balance the recommendation utility and the debias effect, it can be seen that when α=0.7,β=0.1,γ=0.2, the recommendation utility is close to the optimal state, and the debias effect is considerable. Therefore, this paper takes α=0.7,β=0.1,γ=0.2 as the psychological tendency parameter of the proposed model.

### 3.3. Comparative Experiment

**Figure 1:**
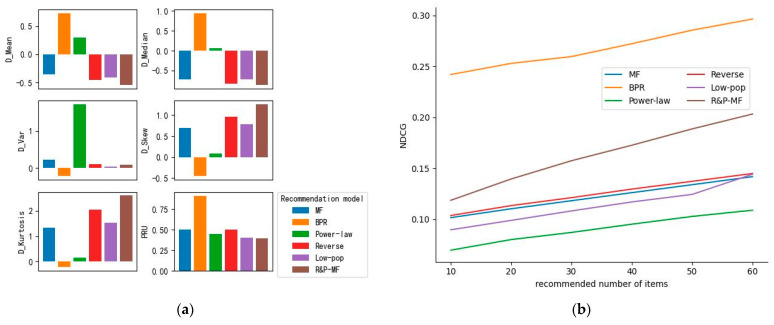
Experimental Settings: λ = 0.02, λ_1_ = 0.04, λ_2_ = 0.04, λ_3_ = 0.002, λ_4_ = 0.03. (**a**) Results of all models (MF, BPR, Power-law, Reverse, Low-pop, and R&P-MF) with D_M^@^60 and PRU as metrics; (**b**) Results of all models (MF, BPR, Power-law, Reverse, Low-pop, and R&P-MF) with NDCG^@^ (10–60) as metrics.

In order to evaluate the performance of the proposed model from two levels of recommendation utility and recommendation bias, the model is denoted as R&P-MF. In this paper, two classical models and three debiasing models are selected for comparison. The comparative experimental results are shown in Figure 1.

(A) MF [46]: Matrix factorization is one of the most commonly used recommendation models due to its good recommendation performance. It reduces the dimension of the rating matrix, obtains the mapping of users and items in the hidden factor space, and uses the latent factor matrix to predict the user rating.
(B) BPR [47]: Pairwise ranking recommendation model based on the Bayesian formula has good performance in dealing with implicit feedback. It assumes that different users have independent preferences and the same user has independent preferences for different items, and constructs user-item interaction behaviors in the form of triples to predict user preferences.
(C) Power-law [32]: A hierarchical test of popularity based on power-law distribution, which assigns weights to the observable ratings of items in the training data, aiming to assign items with low popularity to higher ratings, so that they can obtain higher recommendation rankings in training.
(D) Reverse [32]: Similar to reverse propensity weighting, the original data sample is rescaled according to the popularity of the items to uniformly boost the ratings of low-popularity items.
(E) Low-pop [37]: The items with low popularity are compensated for popularity according to the degree of user preference and the scale of user value. The lower the popularity of the item, the more compensation is obtained.

## 4. Discussion

It can be seen from Figure 1 that BPR has the best recommendation utility and the largest recommendation bias. Both D_Mean and D_Median of BPR and Power-law are positive, indicating that BPR and Power-law tend to recommend more popular items to users on the whole. Compared with BPR, Power-law has more diverse recommended and suggested items than user history items. MF shows the opposite trend to BPR. According to the positive values of D_Skew and D_Kurtosis, Reverse, Low-pop, and R&P-MF increase the recommendation of long-tail items, that is, low-popularity items. R&P-MF has the best performance among the three, and has the best recommendation utility when the bias value reaches the minimum. A larger D_Kurtosis means that more items in the recommendation list are distributed in low-popularity areas. In summary, R&P-MF has the best performance in the comparison model by considering both recommendation utility and debias effect.

The model proposed in this paper focuses on collaborative filtering based on matrix factorization and is a non-pairwise recommendation model. In view of the fact that the pairwise recommendation model such as BPR has strong recommendation utility but at the cost of losing the debias effect, future research will explore the bias problem of the pairwise recommendation model, in order to maintain its good recommendation utility and improve a certain degree of debiasing ability.

Herd mentality causes users to make the same evaluation as others, resulting in the bias of recommendation results. However, the recommendation result will react on the user, resulting in a bad circular effect and affecting the recommendation utility. The model proposed by us effectively alleviates the bias problem and guarantees the recommendation utility.

## 5. Conclusions

### 5.1. Based on Model

The rating bias and popularity bias in recommendation bias exist in data, models, and results, which are important reasons for the unfairness of recommender system process and outcome. Users are influenced by the herd mentality, so that they will produce herd behavior in item selection and rating decisions, and the resulting rating bias will further lead to popularity bias with the training of the recommendation model. In view of this, this paper improves the collaborative filtering recommendation model based on matrix factorization from two stages: data and model. Firstly, k-order parabolic fuzzy distribution is used to fuse the user’s age to adjust the rating, and a similarity measure based on this is constructed to obtain the debiased rating. Secondly, a new matrix factorization loss function is constructed by using the debiased rating as the weight and integrating the continuously increasing flow and popularity of the item, in order to reduce the rating bias and popularity bias. Finally, psychological line and sentiment indicators were introduced as proxy tools to measure user emotion and item popularity, respectively. User emotion and item popularity were mixed to construct user psychological tendency, which was divided into three levels: strong, medium, and weak, and different weights were assigned to different levels to ensure the balance between recommendation utility and debias effect. The model proposed in this paper is compared with other classical models and debiasing models. Experimental results show that the model has good performance in both recommendation utility and debias effect.

### 5.2. Implications

Based on the above research on the rating bias and popularity bias, the important factors affecting commodity sales and user satisfaction can be extracted from them. At the same time, according to the research results of this paper, we can take these recommendations for platforms and merchants to strengthen their commodities’ quality and also pay attention to users’ psychology and preferences.

#### 5.2.1. Strengthen the Quality of High-Popularity Commodities

The recommendation model based on collaborative filtering tends to recommend high-popularity commodities to users. Even when the popularity bias is reduced, the list of recommendation results still contains part of high-popularity commodities. As the “front” of the recommendation platform and the “big head” in the recommendation list, the platform should strengthen the supervision of highly popular commodities, put quality assurance in the first place, take regular sampling inspection strategy for the commodities that have been popular for a long time, pay attention to the user feedback of such commodities, and make corresponding improvements according to the feedback. At the same time, as the source supply of commodities, merchants should check the quality of commodities before they enter the platform, and follow up every key point from the launch to the sale to the feedback, so as to ensure that the commodities with high popularity live up to their name, rather than deceive users under the guise of traffic.

#### 5.2.2. Ensure the Quality of Low-Popularity Commodities

Low-popularity commodities do not receive attention due to their low probability of being recommended. However, the number of low-popularity commodities is far greater than that of high-popularity commodities, accounting for a considerable proportion in the recommendation platform. Increasing the recommendation of low-popularity commodities can bring profits to the platform merchants and bring novel experience to users. Therefore, the quality of low-popularity commodities also needs to be strongly guaranteed. Although the recommendation frequency of low-popularity commodities is far less than that of high-popularity commodities, once they are discovered by users, the quality becomes a decisive factor for whether the commodities will be re-purchased and recommended to social groups by users. At the same time, quality is also the key to commodities’ jump from unpopular categories to frequently purchased commodities, therefore, the quality assurance of low-popularity commodities is undoubtedly crucial.

#### 5.2.3. Pay Attention to Users’ Curiosity

With the increasing number of commodity categories, the number of commodities has exploded on the recommendation platform, and users’ basic needs have been easily satisfied. Some users are not satisfied with the conventional purchase needs or are driven by the psychology of curiosity, which prompts them to turn their eyes to novel and unpopular commodities, but the recommendation mechanism limits the needs of these types of users. Novel commodities and unpopular commodities are often difficult to enter into the public view because of their low frequency of recommendation. In addition to losing their own value, they will also affect the personalized experience of users seeking novelty. Paying attention to users’ curiosity should become a new entry point for platforms to increase profits and retain users. Considering the needs from the perspective of different types of users and taking into account the preferences of different types of users is the key for platforms to improve user satisfaction.

#### 5.2.4. Pay Attention to Users’ Boredom

A long-term recommendation of the same type of high-popularity commodities to users makes it easy to present the user recommendation list with a trend of homogeneity. In the initial state, users will not reject these kinds of commodities due to the popularity and conformity, but with the long-term recommendation, users will become tired of it. Once users start to become tired of such commodities, it will cause unmarketable commodities, affect platform profits, and even cause user loss when users leave the platform. Therefore, the platform should pay attention to the causes and results of users’ psychology. Although the mass sales of high-popularity commodities will bring great profits to the platform, we should not blindly recommend high-popularity commodities of the same type to users. It is important to pay attention to the psychological changes of users. While ensuring the sales volume and traffic of commodities with high popularity, take into account the counter-phenomenon caused by users’ boredom, and make appropriate recommendations to create a good recommendation state.

#### 5.2.5. Pay Attention to Users’ Preferences

The purpose of recommendations is to make the recommendation conform to the real preferences of users. However, with the influence of time, psychology, emotion, and other factors, users’ preferences will have new changes, and even their preferences after the change are quite different from the previous user-commodity interaction records. Therefore, recommendations should always be consistent with user preferences. Depending on the influence of the original data and recommendation mechanism, the recommendation performance of the platform often takes the improvement of the recommendation accuracy as the main evaluation means. Once the user preference changes, the system does not capture these details in time, and the accurate recommendation accuracy becomes the burden of the user. The platform shall pay attention to the real preferences of users and reasonably recommend corresponding commodities according to the change of preferences.

## Figures and Tables

**Table 1:** Information of Movielens dataset.

Dataset		Movielens-100 k	
Users	943	Ratings	100,000
Items	1682	Density	6.3%
Average Popularity	59.45	Age distribution	7~73

**Table 2:** The weights α,β and *γ* of the three intervals of strong, medium, and weak psychological tendency were adjusted, respectively, according to the step size of 0.1, and PRU and NDCG were used as evaluation metrics.

α	β	γ	PRU	NDCG@60
0.8	0.1	0.1	0.4821	0.2073
0.7	0.1	0.2	0.3933	0.2033
	0.2	0.1	0.4777	0.2052
0.6	0.1	0.3	0.3501	0.1838
	0.2	0.2	0.4060	0.1929
	0.3	0.1	0.4677	0.1832
0.5	0.1	0.4	0.2506	0.1546
	0.2	0.3	0.3258	0.1665
	0.3	0.2	0.4042	0.1566
	0.4	0.1	0.4301	0.1245

## Data Availability

Public dataset can be found at https://grouplens.org/datasets/movielens/, accessed on 3 November 2022. The data used to support the findings of this study are available from the corresponding author upon request.
